# Retraction: miR‐365 secreted from M2 Macrophage‐derived extracellular vesicles promotes pancreatic ductal adenocarcinoma progression through the BTG2/FAK/AKT axis

**DOI:** 10.1111/jcmm.18188

**Published:** 2024-02-23

## Abstract

Xin Li, Hao Xu, Jianfeng Yi, Chunlu Dong, Hui Zhang, Zhengfeng Wang, Long Miao, Wence Zhou. miR‐365 secreted from M2 Macrophage‐derived extracellular vesicles promotes pancreatic ductal adenocarcinoma progression through the BTG2/FAK/AKT axis. *J Cell Mol Med*. 2021; 25: 4671–4683. https://doi.org/10.1111/jcmm.16405.

The above article, published online on 03 April 2021 in Wiley Online Library (wileyonlinelibrary.com), has been retracted by agreement between the authors, the journal Editor‐in‐Chief, Stefan Constantinescu, The Foundation for Cellular and Molecular Medicine and John Wiley and Sons Ltd. The corresponding author asked to retract the article as they found that the animal experiments did not support all the conclusions of the paper. Subsequently, an investigation into concerns raised by a third party, revealed inappropriate duplications of images between this and other articles that were either previously published or published later in the same year. Thus, the editors consider the conclusions of this manuscript substantially compromised. Although the authors initially asked for the retraction, they did not agree to the retraction wording.

Xin Li, Hao Xu, Jianfeng Yi, Chunlu Dong, Hui Zhang, Zhengfeng Wang, Long Miao, Wence Zhou. miR‐365 secreted from M2 Macrophage‐derived extracellular vesicles promotes pancreatic ductal adenocarcinoma progression through the BTG2/FAK/AKT axis. *J Cell Mol Med*. 2021; 25: 4671–4683. https://doi.org/10.1111/jcmm.16405.

The above article, published online on 03 April 2021 in Wiley Online Library (wileyonlinelibrary.com), has been retracted by agreement between the authors, the journal Editor‐in‐Chief, Stefan Constantinescu, The Foundation for Cellular and Molecular Medicine and John Wiley and Sons Ltd. The corresponding author asked to retract the article as they found that the animal experiments did not support all the conclusions of the paper. Subsequently, an investigation into concerns raised by a third party, revealed inappropriate duplications of images between this and other articles that were either previously published or published later in the same year. Thus, the editors consider the conclusions of this manuscript substantially compromised. Although the authors initially asked for the retraction, they did not agree to the retraction wording.

